# Computer-assisted lateralization of unilateral temporal lobe epilepsy using Z-score parametric F-18 FDG PET images

**DOI:** 10.1186/1471-2385-7-5

**Published:** 2007-11-02

**Authors:** Ching-yee Oliver Wong, James Gannon, Jeffrey Bong, Christiana O Wong, Gopal B Saha

**Affiliations:** 1 Nuclear Medicine, William Beaumont Hospital, Royal Oak, MI, USA; 2 Radiology, College of Human Medicine, Michigan State University, Lansing, MI, USA; 3 Cranbrook Schools, Cranbrook Educational Community, Bloomfield Hills, MI, USA; 4 Nuclear Medicine, Cleveland Clinic Foundation, Cleveland, OH, USA

## Abstract

**Background:**

To evaluate the use of unbiased computer-assisted lateralization of temporal lobe epilepsy (TLE) by z-score parametric PET imaging (ZPET).

**Methods:**

38 patients with histologically proven unilateral TLE due to pure hippocampal sclerosis, referred for pre-surgical PET evaluation of intractable seizure over a 5-year period, were included. The F-18 FDG images were oriented along temporal long axis and then transformed into ZPET images on a voxel by voxel basis. Multiple regions of interests (21 in total) were placed on cortical, subcortical and cerebellar structures on twenty-eight out of 38 patients with totally seizure-free (class I) outcome. Paired t-tests with Bonferroni correction were used to determine the location of the most asymmetric regions as variables for subsequent discriminant analysis of the entire group of the patients.

**Results:**

The computer program identified the anterior half of the temporal lobe (p < 0.0005) and thalami (p = 0.021) as the most asymmetric regions in TLE patients with Class I outcome. Discriminant analysis using z-scores from a total of 8 ROIs (in 4 pairs) on these structures correctly lateralized thirty-seven out of 38 (97%) patients (sensitivity = 94%; specificity = 100%). The only false localization came from a patient with equivocal z-scores on the temporal lobes and this patient turned out to have poor outcome.

**Conclusion:**

The computer-assisted lateralization of TLE using ZPET provides an accurate, fast and objective way of seizure evaluation.

## Background

The interictal state of temporal lobe epilepsy (TLE) is characterized by decreased uptake due to hypometabolism on F-18 fluorodeoxyglucose (FDG) positron emission tomography (PET) images. Spanaki et al. suggested that seizures might have a reversible effect on brain areas connected with, but remote from, the epileptogenic cortex [1]. Vinton et al found patients with medically refractory temporal lobe epilepsy to have a better surgical outcome when a greater proportion of the hypometabolic volume was resected [2]. Choi et al noticed that extratemporal cortical hypometabolism outside the seizure focus, in particular hypometabolism in the contralateral cerebral cortex, might be associated with a poorer postoperative seizure outcome in TLE. These regions might represent underlying pathology that was potentially epileptogenic [3]. Such results indicate that discerning the presence and the exact extent of hypometabolism is clinically significant in planning surgery and predicting outcome. Thus, automatic unbiased techniques to better elucidate regional metabolic abnormalities are important to the treatment of patients with temporal lobe epilepsy.

Routine interpretation of PET images to evaluate hypometabolism involves visual assessment of images normalized to the region of maximal uptake. Color scales or continuous gray scales are used to determine qualitative differences in metabolism. Unfortunately, besides human observer variability, factors such as medications and external stimuli yield variability in glucose metabolism, which affects the maximal pixel count between patients and temporally within the same patient. Traditional analysis also involves comparison of homologous regions, which implicitly assumes a relatively normal contralateral side. Wong et al [4] proposed a method using individual subject's z-map parametric statistical imaging. The method utilizes an individual's mean cerebral glucose metabolism and indexes the changes relative to the mean rather than maximum pixel intensity. Such a map models each pixel in terms of the standard deviation from the mean glucose metabolism of the particular subject. It does not require the use of a group of normal subjects for comparison and assumes the identical nature of all human brains.

The average intra-subject coefficient of correlation between the individual normal subjects' right and left hemispheric metabolism has been shown to be 80% [5]. The averages of the inter-subject coefficients of correlation of a particular region of normal subjects' metabolism against their respective global means of all regions from each individual subject are also around 80%, which are not significantly different between the hemispheric intra-subject's variability [5]. Besides, the calculation of the global mean will not be significantly affected by as much as 8% of brain regions being abnormal [4]. Thus, despite the potential normal brain heterogeneity, the comparison to the global mean would not differ from the traditional side by side comparison used for unbiased clinical interpretation of brain PET images.

The purpose of this study is to retrospectively evaluate the use of totally unbiased automatic computerized determination of the lateralization and the extent of epileptic foci utilizing the z-map parametric statistical imaging.

## Methods

### Patients

Interictal PET images from 38 patients with surgically proven unilateral temporal lobe epilepsy due to pure hippocampal sclerosis referred for pre-surgical PET evaluation of intractable seizure over a 5-year period at mean age of 31 were analyzed. All patients had extensive clinical, EEG, neuro-psychologic and MRI evaluation. In 18 patients, the seizure focus was found on the left temporal lobe. The other 20 patients had right side temporal focus. Twenty-eight of the 38 patients had seizure free (Engel Class I [6]) outcome after surgery.

### Scintigraphic methods

All patients received an average dosage of 10 mCi (370 MBq) of F-18 FDG in a fasting state. EEG monitoring was performed shortly before the tracer injection and during the uptake period of the F-18 FDG. PET image acquisition was begun about 45 minutes after injection. The PET camera was a Posicam 6.5 (Positron, Houston, TX) and data were acquired in a 256 × 256 matrix with a pixel size of 1.7 mm, using a uniform attenuation coefficient of 0.096 cm^-1^. The images were re-oriented along the temporal axis and zoomed to a final pixel size and slice thickness of 3.4 mm.

### Image Analysis

A voxel by voxel transformation of the PET image into a z-score parametric PET image was performed using PV-WAVE ADVANTAGE (Visual Numerics, Boulder, CO) using the methods described below. The mean and standard deviation of pixels whose counts were greater than 50% of the maximal count were calculated. Then the whole volume of brain image was transformed into z-scores on a pixel by pixel basis using the formula [4]:

***Z(x, y, z) = { I(x, y, z) - μ }/σ***

where μ and σ are the respective mean and standard deviation of the pixel counts. The 50% of maximum count was chosen to exclude ventricles and white matter from being involved in the calculations. Since at least 20,000 pixels are used in the calculations, the pixel distribution is essentially Gaussian and a small percent of outlier values due to seizure focus will not significantly affect the results [4].

**Figure 1:**
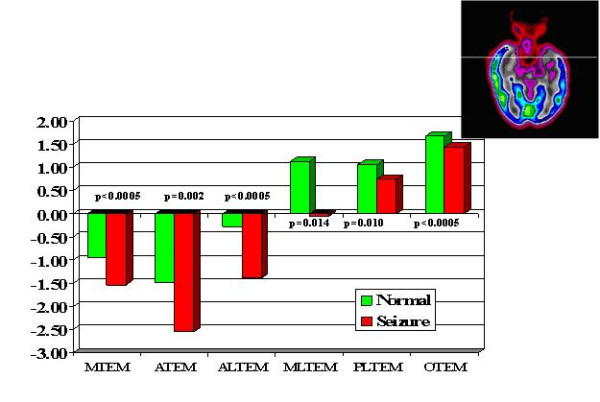
Temporal lobe z-scores. TEM = temporal, M = mesial, A = anterioir, AL = anterolateral, PL = posterolateral, O = occipital.

**Figure 2:**
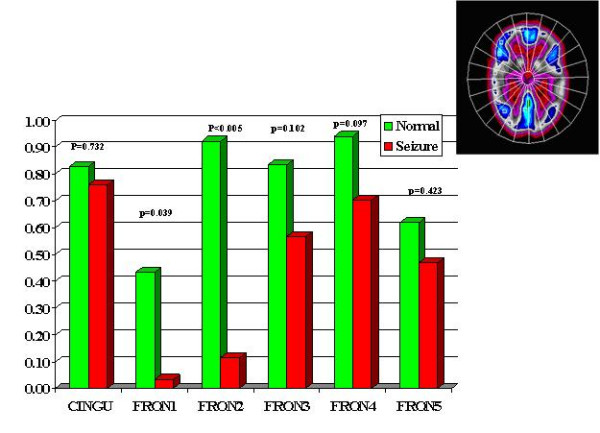
Frontal z-scores. CING = cingular, FRON = frontal: 1 to 5 from anterior to posterior.

**Figure 3:**
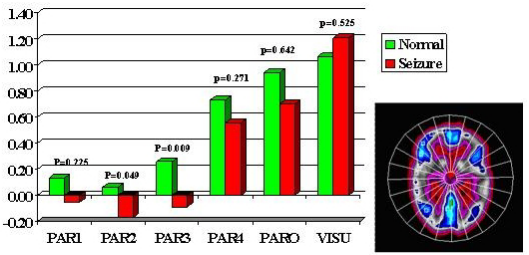
Parietal and occipital z-scores. PAR = parietal: 1 to 4 from anterior to posterior; PARO = parieto-occipital; VISU = visual cortex.

**Figure 4:**
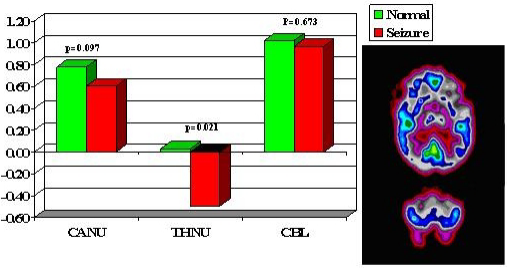
Subcortical and cerebellar z-scores, CANU = caudate, THNU = thalamus, CBL = cerebellum.

**Figure 5:**
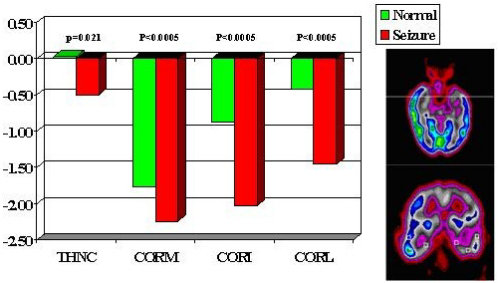
ROIs used for discriminant analysis. THNC = thalamus; COR = coronal: M, I, L represent mesial, inferior, lateral temporal lobe.

Multiple regions of interest (ROIs) were then placed on the z-map images guided by an automatic sectorial gird generated by computer [5]. A total of 21 pairs of ROIs were placed on transaxial slices rotated along the temporal long axis in anterior frontal (AF), posterior frontal (PF), parietal (PA), occipital-parietal (OP), anterior temporal (AT), posterior temporal (PT) regions, caudate nucleus (CN), thalamic nucleus (TN) and cerebellum (CB). There were six temporal regions generated labeled as: mesial, anterior, anterolateral, mesioateral, posterolateral and occipitotemporal regions (Fig. 1). There were six frontal regions: cingulate gyrus, and 5 other frontal regions labeled 1 to 5 from anterior to posterior frontal lobe (Fig 2). There were 4 parietal regions labeled as 1 to 4 from anterior to posterior area, 1 parietooccipital region and 1 visual cortical region (Fig 3). Lastly, there was one pair of ROIs on each of the cerebella, caudate and thalamic nuclei (Fig. 4). In addition, 4 pairs of ROIs on the coronal slice through the mid anterior temporal region, which covered the most hypometabolic areas of TLE on the temporal lobes and thalami, were used for discriminant analysis in predicting seizure focus (Fig. 5).

### Statistical Analysis

The asymmetry in FDG metabolism was evaluated by comparing differences between homologous regions in the subset of patients (28 out of total 38) with totally seizure free (class I) outcome. Paired t-tests with Bonferroni correction were performed to determine the location and hence the extent of the most asymmetric regions on metabolism (Figs. 1 to 5). Discriminant analysis was performed against the dichotomous results of seizure focus using z-scores of the 4 pairs of ROIs from the temporal and thalamic regions (Fig. 5, Table 1) as independent variables to lateralize the seizure site on all the 38 patients.

**Table 1:** Results of Discriminant Analysis, showing high discriminant power of z-score PET (ZPET) in lateralization of seizure focus (p < 0.000001).

Total = 38 Patients	**Abnormal ZPET**	**Normal ZPET**
**Seizure side (18)**	17/18 (94%)	1/18 (6%)*
**Normal size (20)**	0/20 (0%)	20/20 (100%)

*This patient had equivocal z-score on temporal lobe and suffered from poor surgical outcome.

## Results

All ZPET images analyzed were truly interictal based on the EEG and clinical monitoring of seizure activity during the uptake phase of F-18 FDG before PET imaging. The computer program identified the anterior half of the temporal lobe (p < 0.0005) and thalami (p = 0.021) as the most asymmetric regions in TLE patients with Class I outcome. Discriminant analysis using z-scores from the most asymmetric regions correctly lateralized thirty-seven out of the 38 (97%) of patients (sensitivity = 94%; specificity = 100%). The only false localization came from a patient with equivocal z-scores on the temporal lobes and this patient had poor outcome. The results suggested also extension of functional abnormality outside the temporal lobe into the ipsilateral thalamic, frontal and parietal areas (Figs. 2, 3, 4). On the other hand, the majority of the archeocortical structures like visual cortex, cingulum, sensorimotor, caudate and cerebellum were not affected (Figs. 2, 3, 4).

## Discussion

Hypometabolism, manifested by decreased glucose uptake on FDG-PET scans of interictal seizure patients, is correlated with the postulated epileptogenic zone and adjacent surrounding areas. Takaya et al [7] found that prefrontal hypometabolism led to deficits on the Modified Wisconsin Card Sorting Test and showed more preservative errors by the frequent seizure group than the rare seizure group. Joo et al [8] indicated that there was restoration of frontal lobe glucose metabolism in patients with good seizure control after temporal lobe epilepsy surgery. This suggests the functional nature of extratemporal metabolic abnormality noted in the current study.

Vinton et al [2] studied the relationship of FDG PET statistical parametric mapping by quantifying a volume of hypometabolism and co-registering the PET with MRI to study the relationship of the amount of hypometabolic tissue removed during temporal lobe resection and seizure outcome. They found the more hypometabolic region resected, the better the outcome. Thus accurate unbiased computer determination in a consistent manner to reliably localize temporal lobe hypometabolism will aid in determining the surgical field of resection and improve patient outcome. The initial work by Wong et al [4] used the strict classification of "good outcome" as seizure free (Class I) outcome and showed statistical parametric mapping z-map to be an accurate method to evaluate the correlation between the degree of hypometabolism and seizure outcome. The threshold z-score of -1.5 found by Wong et al [4] for the discriminating power of ZPET in predicting surgical outcome was important in visual identification of areas with functional (metabolic) abnormality due to seizure. The same method was employed in the current study to evaluate by computer, without visual input, for the total extent of functional metabolic cortical abnormality due to TLE caused by a homologous pathology of pure temporal lobe sclerosis. The discriminant analysis again reiterated the prior observation [4] that equivocal distinction of z-scores of metabolism by FDG PET between the temporal lobes was associated with poor outcome (Table 1). The diagnostic results were similar to other studies using automatic artificial neural network [9].

The prognostic value of exact location of hypometabolism measured by PET within the temporal lobe is still debating. Some studies suggested that patients with mesial temporal hypometabolism on PET might have a higher probability of becoming seizure free postoperatively than did patients with hypometabolism in other parts of the temporal lobe [10,11]. However, other studies indicated that lateral temporal lobe [12] or temporal pole hypometabolism [13] were better predictors of a seizure-free postoperative outcome. Most of the prior studies included patients who had different etiologies, such as mesial sclerosis, tumor and dysplasia. This prognostic controversy has been partly addressed by Lee et al [14] using relative asymmetric index in patients with pure temporal sclerosis.

The patients recruited for the study were highly selected surgical candidates with pure temporal sclerosis. All the available tests (MRI, PET, EEG) and clinical and neuropsychological evaluation converged to the same conclusion before surgery was performed. Thus the numbers with poor surgical outcome might be small or the analysis might be biased towards good prognostic implications. Further studies on both surgical and non-surgical candidates using long term follow up are required to further address the true prognostic implications of the location, extent and severity of hypometabolism measured by PET.

It was interesting to note the computer findings in current study about the involvement of ipsilateral thalamus in TLE. It might be due to sensory deafferentation from the severity or chronic nature of TLE. As Choi et al [3] had pointed out, thalamic hypometabolism was associated, but not independently, with a higher likelihood of postoperative seizures [3]. Contralateral thalamic involvement might be secondary to extratemporal or temporal pathology. This was outlined by Newberg et al [15] in their study of patients with temporal lobe epilepsy. They found that the thalamic metabolic asymmetry, particularly in the reverse direction (contralateral) to that of the temporal lobe asymmetry, was associated with a poor post-surgical outcome compared with no or matched asymmetry. Thus the current method of automatic unbiased computerized determination of the extent and severity of brain hypometabolism is important in evaluating epileptic patients and selecting optimal candidates for surgical intervention.

Statistical parametric mapping removes inter-observer variations as shown by Vinton et al [2] and Wong et al [4]. The study successfully utilized totally unbiased computer assisted statistical parametric analysis using z-scores for lateralization to find significant metabolic differences among various brain regions from the functional effects of epilepsy due to pure temporal lobe sclerosis and localized them in a manner which correlated well in patients having a good surgical outcome. Since the study was designed for unilateral disease using computer for side to side comparison of z-scores, the methodology may not be applicable or may be limited in bilateral disease. Further studies are needed from proven bilateral cases to address this difficult issue, which include at least some determination of a potential threshold z-score value based on clinical prognosis[4].

As the effects of epileptic focus are usually contiguous (by neuronal connections) and the main focus of the study is to evaluate to the extent of functional abnormality from pure temporal lobe epileptic focus, the traditional ROI sampling guided by automatic individualized grid used in the current study will be a sufficient sampling method for detecting the extent of the abnormality. The more precise method of statistical probabilistic anatomical mapping (SPAM) would be useful if one want to know the exact focus of activation or abnormality, which was first used in MRI [16] and recently also in functional imaging such as single photon emission computed tomography (SPECT) [17] and PET [14]. However, considering the objective of our current study, the simplicity of the method used, the resolution of the PET camera and the Gaussian nature of the tracer distribution, the current methodology will be a good approximation to the probabilistic anatomical mapping. The automatic computer method helped to determine the location, severity and extent of functional brain abnormalities due to temporal lobe seizure, unbiased by any human input.

## Conclusion

The results show significant metabolic differences between homologous brain regions determined by computer assisted z-score lateralization that accurately identify regions and the extent of hypometabolism associated with temporal lobe seizures. The computer-assisted lateralization of TLE using ZPET provides an accurate, fast and objective way of seizure focus and extent evaluation.

## Abbreviations

EEG, Electroencephalography; FDG, F-18 fluorodeoxyglucose; MRI, Magnetic resonance imaging; PET, Positron emission tomography; ROI, Region of interest;

TLE, Temporal lobe epilepsy; SPAM, Statistical probabilistic anatomical mapping;

SPECT, Single photon emission computed tomography; ZPET, Z-score parametric PET imaging.

## Competing interests

The author(s) declare that they have no competing interests.

## Authors' contributions

CYOW conceived of the study, participated in its design and coordination, performed computer programming and statistical analysis and involved in manuscript writing. JG and JB carried out data collection and literature search and involved in manuscript writing. COW helped in data collection, performed literature search and prepared illustrations. GBS participated in data analysis, study coordination and manuscript writing. All authors read and approached the final manuscript.

## Pre-publication history

The pre-publication history for this paper can be accessed here:


